# Supplementary material for: Association between oral microbiome and seven types of cancers in East Asian population: a two-sample Mendelian randomization analysis
Source: Front Mol Biosci. 2023 Nov 21;10:1327893. doi: 10.3389/fmolb.2023.1327893 (PMC10702768; doi:10.3389/fmolb.2023.1327893)
Supplement: Supplementary file 1 [file DataSheet1.ZIP › Supplementary Materials/MR plots of saliva/saliva═╝/Pancreatic cancer/pheno.3681_to_pancreatic cancer_leave_one_out.pdf]

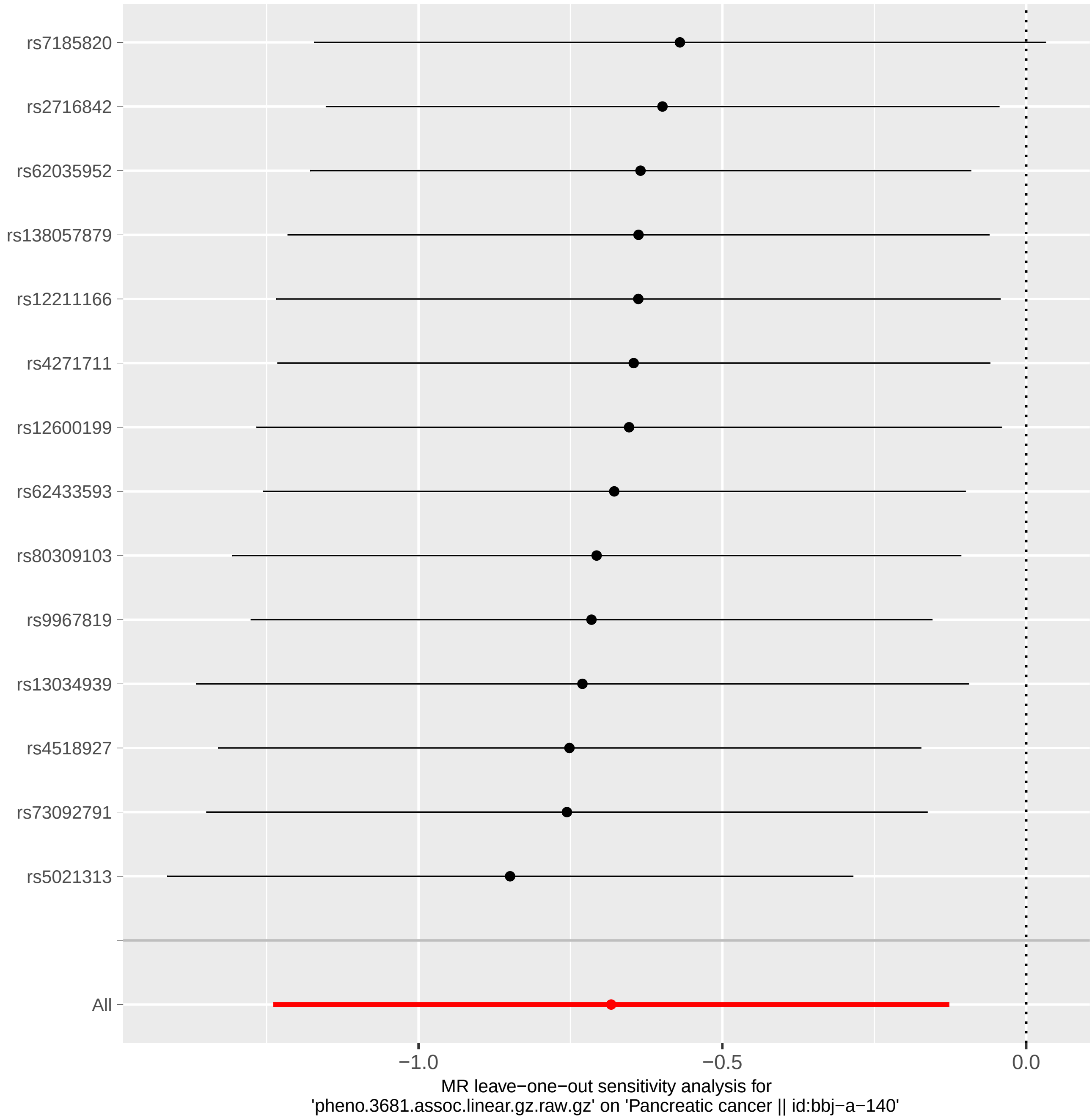
rs7185820
rs2716842
rs62035952
rs138057879
rs12211166
rs4271711
rs12600199
rs62433593
rs80309103
rs9967819
rs13034939
rs4518927
rs73092791
rs5021313
All
−1.0
−0.5
0.0
MR leave−one−out sensitivity analysis for
'pheno.3681.assoc.linear.gz.raw.gz' on 'Pancreatic cancer || id:bbj−a−140'
